# How Could the Ethical Management of Health Data in the Medical Field Inform Police Use of DNA?

**DOI:** 10.3389/fpubh.2018.00154

**Published:** 2018-05-29

**Authors:** Gaelle Krikorian, Joëlle Vailly

**Affiliations:** UMR8156 Institut de recherche interdisciplinaire sur les enjeux sociaux, Paris, France

**Keywords:** DNA, police, ethics, genetic technologies, criminal investigations

## Introduction

Various events paved the way for the production of ethical norms regulating biomedical practices, from the Nuremberg Code (1947) —produced by the international trial of Nazi regime leaders and collaborators—and the Declaration of Helsinki by the World Medical Association (1964) to the invention of the term “bioethics” by American biologist (1). The ethics of biomedicine has given rise to various controversies—particularly in the fields of newborn screening (2), prenatal screening (3), and cloning (4)—resulting in the institutionalization of ethical questions in the biomedical world of genetics. In 1994, France passed legislation—commonly known as the “bioethics laws”— to regulate medical practices in genetics. The medical community has also organized itself in order to manage ethical issues relating to its decisions, with a view to handling “practices with many strong uncertainties” and enabling clinical judgments and decisions to be taken not by individual practitioners but rather by multidisciplinary groups drawing on different modes of judgment and forms of expertise (5). Thus, the biomedical approach to genetics has been characterized by various debates and the existence of public controversies.

In the judicial sphere, the situation is very different. Since the end of the 1990s, developments in biomedical research have led to genetic data being used in police work and legal proceedings. Today, the forensic police are omnipresent in investigations: not just in complex criminal cases but also routinely in cases of “minor” or “mass” delinquency. Genetics, which certainly receives the most media coverage among the techniques involved (6), has taken on considerable importance (7). However, although very similar techniques are used in biomedicine and police work (DNA amplification, sequencing, etc.), the forms of collective management surrounding them are very different, as well as the ethico-legal frameworks and their evolution, as this text will demonstrate.

## Nature of the information and genetic data produced in the police sphere

In police work in France, data produced by DNA are currently compiled and used in two different ways: first, to create files on individuals in the FNAEG or *Fichier national automatisé des empreintes génétiques* (national automated DNA database) and, second, in order to obtain information about perpetrators of crimes (their appearance, their origin, their kinship links to other individuals).

Police use of DNA has been allowed in France since the 1998 law providing for the creation of the FNAEG. A DNA profile corresponds to a “specific individual alphanumeric combination” (8) that is the numerical encoding of analysis of DNA segments. This profile is the result of analysis of DNA fragments using genetic markers. This analysis can be carried out on a minute amount of genetic material (saliva, blood, sperm, hair, contact, etc.). It identifies the presence of sequences specific to an individual that differentiate them from any other person (with the exception of an identical twin) but that are not supposed to provide any phenotypical information (about appearance, geographical origin, or diseases)^1^. Such profiles therefore make individuals “identifiable in their uniqueness” (9). During investigations, DNA is collected from suspects or unidentified stains left on crime scenes or people and the results of this analysis are entered into the database. Identification through the FNAEG was originally restricted to a limited number of crimes—those of a sexual nature, as part of the law relating to the prevention and punishment of sexual crimes and the protection of minors. This remit has progressively been extended to include the vast majority of crimes and offences^2^, leading to the routine use of DNA in investigations^3^. As a result of this evolution, there has been a substantial increase in the number of persons with files in the FNAEG: more than 3 million as of late 2015^4^.

New techniques have also emerged in recent years. It is now possible to obtain indications about an individual's physical appearance based on a sample of his/her DNA (10, 11): the analyses in question provide statistical information on eye, hair, and skin color, etc. These techniques are more exploratory and aim not to match DNA with an identity by comparison but to determine the characteristics of the perpetrator of a crime. These data result from analysis of several dozen DNA markers that, unlike the FNAEG's data, are selected deliberately so that they can provide information about a person's physical appearance. They are therefore aimed at “generating a suspect” [(12): 427] but because the information about this person's features are incomplete (e.g. a person with blue eyes, fair skin, light brown hair, and of European “bio-geographical” ancestry), they define “target populations of interest” to guide police investigations (13). Several private and public laboratories in France now produce what professionals often refer to as “DNA photofits”: it is estimated that several dozen such analyses have been carried out since 2014 as part of investigations.

## How is this framed legally, politically, and ethically?

The legal framework surrounding how the police and justice system use DNA analysis was devised to follow the creation of the FNAEG. For this reason, and in order to defuse fears and criticisms, the law only allows analyses using “non-coding” DNA so as to meet the initial objective of allowing identification without providing information about individuals. French law only provides the police DNA for identification purposes “within the framework of investigative measures or the preparation of a case during a judicial proceeding”^5^, in cases of missing persons^6^, or, more recently, in the context of familial searches to allow “searches for persons directly related to [an] unknown person” who has left a stain at a crime scene (i.e. without determining phenotype)^7^.

Concerning the so-called “DNA Photofit” technique, in June 2014, France's highest court, the Court of Cassation, ruled admissible an expert report charged with providing “all useful elements relating to the suspect's visible morphological characteristics” based on stains collected after a rape in an investigation into a series of sexual assaults in Lyon between October 2012 and January 2014. The Court of Cassation's authorization of this practice in DNA analysis was the first in France. For judges and prosecutors, there is now set a legal precedent allowing them to authorize “DNA Photofits” when they consider this could help an investigation.

In legal terms, the emerging of new technical possibilities and their practical use create conflicting and parallel regimes. On one hand, “DNA Photofits” do not correspond to the legal frameworks devised in the 1990s: it does not provide identification, per se, but is rather an “assistance to the investigation,” as it uses coding DNA. One another hand, as science evolves, the law is falling out of step with the technical and scientific reality. New knowledge shows that some of the markers used by the FNAEG may in fact allow further information to be obtained about people regarding their predisposition to certain diseases, their genetic pathologies, and their “ethnic origin” (by continent or sub-continent)^8^. Moreover, whereas at the FNAEG's inception it was considered unacceptable for the police to use medical information, certain professionals in police or justice now recognize that this information (whether genetic or not) can be useful in investigations (providing information about wanted persons' need for medication or healthcare, or about their physical appearance, etc.). Although there are no changes in the legal framework on this matter, the idea is spreading and the red line is, to some extend, and for some of the professionals, fading.

It is thus obvious, that police uses of DNA data providing information about individuals' characteristics raise novel politic-ethical issues [(17): 520; (18)]. In particular, it brings into play the issue of what constitutes private data (19)—for certain geneticists, where “DNA Photofits” are concerned, externally visible characteristics do not fall into this category because they are visible (11). Generally, as stated by some professionals during interviews, the question is “to know until where to go. And where to stop. “Regarding the FNAEG and French law, in a case heard in June 2017, the European Court of Human Rights (ECHR) ruled that “interference with the applicant's right to respect for his private life had been disproportionate”^9^. The ECHR judgment ruled against France and underscored that French law regarding DNA date storage should be differentiated “according to the nature and seriousnessness of the offence committed”^10^.

In Germany, a contradictory dialogue between experts took place regarding Forensic DNA Phenotyping revealing public and political debate on the matter (20). In France, despite the stakes involved and the spread of new usages of DNA techniques, no public debate has emerged in recent years concerning new uses of DNA in police work. In 2008, a private analysis laboratory offering indicative geo-genetic tests (*tests d'origine géo-génétique* or TOGG) providing information about individuals' origin based on their DNA, sparked a media debate that problematized the issue (21), however the controversy soon died down. A few years later, Ministry of Justice instructions to judges and prosecutors discouraged the use of this technique, with no further debate. Since then, although the Court of Cassation's 2014 decision opened up the possibility of using an unprecedented practice, this has not generated any public debate or controversy. “DNA Photofits” have received some media coverage^11^, but this has mainly been to underscore the technical process involved, echoing the fiction conveyed by television series that have made the use of genetic techniques in criminal investigations seem commonplace and particularly efficient. Our sociological fieldwork has revealed, however, that there was organized debate among judges and prosecutors between 2013 and 2014. At the time the investigating judge who had for the first time ordered the analysis of the suspect's visible morphological characteristics referred the case to the examining chamber himself, to obtain a verdict on whether the expert report he had requested was legal. Although the examining chamber approved the report, the public prosecutor brought the issue before the Court of Cassation—the highest legal authority in France— in order to ensure the final nature of the decision. The Court of Cassation ruled that a judge could have recourse to such analyses. Following this verdict, several bodies consulted by the Ministry of Justice^12^ provided opinions underscoring the need for this technique to be written into and regulated by the law. This has not been implemented to date. After being authorized for several years under a temporary protocol, familial searches allowing “genetic proximity testing” (22) were written into law in 2016. However, the Court of Cassation's judgment on DNA analysis to provide “all useful elements relating to a suspect's visible morphological characteristics” has not been brought up for parliamentary debate to be included in the law. There has been no political management of the question at State level, nor has the issue been included in the general debate organized by the National Consultative Council of Ethics (Comité Consultatif National d'Ethique) in 2018 regarding the revision of laws on bio-ethics.

## Conclusion

The use of these new technological and scientific techniques plays a significant role in guiding how we engage with the world (23), just as it redefines the production of identity translated into information (24) and structures the way sensitive information about individuals is used and circulated. Despite these stakes, and the initial caution that surrounded the creation of the national automated DNA database, it has not gone hand-in-hand with collective political and ethical debate. This raises questions about the conditions for the existence or for the absence of political controversies that call for further sociological investigations about the framing of the issue and the social and political logics at play.

As the uses of these techniques are developing in police practices, this absence of collective management of the issue refers the professional to forms of local arbitration. Our fieldwork has shown that they are aware that these practices raise issues and therefore devise ethical frameworks for their own use of DNA. As a consequence, in this field, as it is the case in others, ethical issues are addressed in a fragmented manner as endogenous ethical frameworks are “cobbled together” by professionals as a function of their practices and needs. Each institution, laboratory, and in some cases each individual, is crafting a frame and a perimeter of limits to what can be done according to their understanding and appreciation of the legal setting, the practical utility of actions and the ethical constraints perceived.

The ECHR's recent ruling against France regarding the FNAEG may force lawmakers to reach a verdict on this issue, thereby triggering what seems like necessary public debate on forensic use of DNA. The new possibilities provided by genetic technologies point to the need for promoting dialogue among the various professionals using this technology in police work (forensic teams and geneticists working with them, police investigators, private laboratories, prosecutors, judges, etc.), but also with healthcare professionals—who already have experience of the institutionalized management of ethical considerations relating to their practices in genetics—and, more broadly, in society as a whole.

## Author contributions

GK is the main contributor. JV is the head of the research programme and collaborated to the writing of the article.

### Conflict of interest statement

The authors declare that the research was conducted in the absence of any commercial or financial relationships that could be construed as a potential conflict of interest.

## Footnotes

^1^The Order of 10 August 2015 increased the number of markers analyzed to 21—policemen and analysis laboratories had three years to comply with this new requirement.

^2^Act n°98-468 of 17 June 1998 relative to the punishment of sexual crimes and the protection of minors introduced article 706-54 into the Code of Criminal Procedure making provision for the creation of an automated national database to centralize the DNA profiles of persons convicted of offences of a sexual nature. The remit of the database was then extended on several occasions. In 2001, it included serious crimes against persons. In 2003, the law on internal security extended it to persons convicted of or implicated in crimes and offences against persons or property.

^3^Collecting DNA samples in investigations is now the rule. An *ad hoc* body of staff has been trained over the past fifteen years that almost systematically processes crime scenes.

^4^This figure was provided to the French Parliament by the Ministry of the Interior following a question by parliamentarian Sergio Coronado (member of the “Ecologist” parliamentary group) (http://questions.assemblee-nationale.fr/q14/14-79728QE.htm).

^5^Art. 16.11 of the Civil Code.

^6^Art. 26, Domestic Security Guidance and Planning Act n° 95-73 of 21 January 1995.

^7^This possibility was written into law in 2016 in article 796-56-1-1 of Act n° 2016-731 of 3 June 2016 strengthening provisions for the fight against organized crime, terrorism, and their financing, and improving the efficiency and guarantees of the criminal procedure.

^8^For example, according to a study by the Telethon Institute of Genetics and Medicine, D2S1388, one of the markers used by the FNAEG, plays a determining role in the transmission of pseudohyperkalaemia, a rare genetic disease (14). In 2011, a publication by Chinese researchers highlighted the association between marker D21S11-28.2 and coronary heart disease (15). A team of Portuguese researchers (16) has developed an online calculator capable of correlating certain markers used in the FNAEG's DNA samples with individual affiliation to population groups (Sub-Saharan Africa, Eurasia, East Asia, North Africa, Near East, North America, South America, and Central America).

^9^Case of Aycaguer V. France, 22 June 2017, 8806/12, ECHR, Court (Fifth Section).

^10^See legal summary, available here: http://hudoc.echr.coe.int/eng?i=002-11703.

^11^A search conducted on the press database *Europresse* for the period 2010 to 2018 brought up around 70 pieces published mentioning the terms “DNA Photofits” or “Genetic photofits”.

^12^These bodies were the *Commission nationale consultative des droits de l'homme* (CNCDH – National consultative committee on human rights) and the approval committee for people authorized to conduct identification procedures using DNA profiles in the context of legal proceedings or the extrajudicial procedure for identifying deceased persons.
